# Polymer-Based Prodrugs: Improving Tumor Targeting and the Solubility of Small Molecule Drugs in Cancer Therapy

**DOI:** 10.3390/molecules201219804

**Published:** 2015-12-04

**Authors:** Sonja Dragojevic, Jung Su Ryu, Drazen Raucher

**Affiliations:** Department of Biochemistry, University of Mississippi Medical Center, 2500 North State Street Jackson, MS 39216, USA; sdragojevic@umc.edu (S.D.); jryu@umc.edu (J.S.R.)

**Keywords:** polymer based prodrugs, anticancer drugs, macromolecules, drug delivery, cancer therapy, genetically engineered biopolymers, synthetic and natural polymers

## Abstract

The majority of anticancer drugs have poor aqueous solubility, produce adverse effects in healthy tissue, and thus impose major limitations on both clinical efficacy and therapeutic safety of cancer chemotherapy. To help circumvent problems associated with solubility, most cancer drugs are now formulated with co-solubilizers. However, these agents often also introduce severe side effects, thereby restricting effective treatment and patient quality of life. A promising approach to addressing problems in anticancer drug solubility and selectivity is their conjugation with polymeric carriers to form polymer-based prodrugs. These polymer-based prodrugs are macromolecular carriers, designed to increase the aqueous solubility of antitumor drugs, can enhance bioavailability. Additionally, polymer-based prodrugs approach exploits unique features of tumor physiology to passively facilitate intratumoral accumulation, and so improve chemodrug pharmacokinetics and pharmacological properties. This review introduces basic concepts of polymer-based prodrugs, provides an overview of currently emerging synthetic, natural, and genetically engineered polymers that now deliver anticancer drugs in preclinical or clinical trials, and highlights their major anticipated applications in anticancer therapies.

## 1. Introduction

Cancer is one of the major fatal diseases, with current treatments of limited therapeutic efficacy, despite great progress in a range of approaches to tumor eradication, including chemotherapy. The mechanism of action of anti-cancer drugs relies on arresting the cell cycle and rapidly killing all proliferating cells. This killing includes non-cancerous cells, such as bone marrow, gut epithelia, lymphatic system, red blood cells and hair follicles. One of the main challenges in current chemotherapeutic treatments is drug toxicity to healthy organs due to lack of selectivity. To achieve therapeutic efficacy, it is often necessary to administer high doses of drugs because of the drugs’ physicochemical properties, such as short circulation time in the blood plasma. The problems associated with the low specificity and poor pharmacokinetics and pharmacodynamics of chemotherapeutic drugs are well known; however, another frequently encountered difficulty is their poor aqueous solubility, which restricts bioavailability.

Intractable problems besetting drug solubility in aqueous solutions have imposed serious clinical constraints on ascertaining effective therapeutic plasma concentrations so as to achieve appropriately targeted pharmacological responses. At present, ensuring the presence of a therapeutic concentration of poorly soluble drugs in systemic circulation requires the administration of high concentrations of potent chemotherapeutics, with their solubility related to particle size. Smaller particles have surface area to volume ratio increases, which allows greater interaction with the solvent [1]. The most common methods for reducing particle size are micronization and nano suspension [2]. Micronization relies on mechanical stress, using a technique such as spray drying to break up drug aggregates. Nano suspension relies on forcing the drug suspension under pressure through a nano aperture valve, resulting in drug nanoparticles that can then be stabilized by surfactants. Other new techniques for reducing particle size are also forthcoming, such as sonocrystalization, supercritical fluid processes and wet milling [3,4].

Despite successes within each of these approaches, scientists continue to encounter major concerns related to particle size reduction. These concerns largely arise owing to the strong tendency for particles to agglomerate subsequent to size reduction. Such agglomerates are likely to reduce the drug’s therapeutic activity.

A drug’s chemical structure and property determine possible approaches for improving their solubility in aqueous solutions. As drugs may be weakly acidic, weakly basic or hydrophobic, appropriate approaches for increasing their rate of solubility may include pH adjustment, chemical modification, micellar solubilization, hydrotropy, solid dispersion, complexations, microemulsions and co-solvency [5]. Each method possesses advantages, but may also introduce challenges in formulation design, with consequent limitations in therapeutic efficacy. For example, drugs that can be dissolved in water by adjusting their pH may precipitate in blood upon intravenous administration, due to the strong buffering capacity of blood, with its pH between 7.2 and 7.4 [6], leading to emboli.

Similarly, another promising approach for improving drug solubility relies upon mixing hydrophobic drugs with co-solutes, which can open more hydrophilic moieties and so facilitate solubility. However, co-solutes often exert more toxic and immunogenic effects, limiting their effective use at doses required for eradicating cancer cells.

One typical example is Paclitaxel, a microtubule stabilizing agent that has been deemed one of the most significant advances in anticancer therapy [7,8]. This promising anti-cancer agent, however, has poor water solubility—but it does dissolve in organic solvents. Among the formulations used to increase Paclitaxel solubility for intravenous administration is a vehicle containing a blend of ethanol and Chremophor, or polyethoxyated castor oil. The side effects of Chremophor, however, include neurotoxicity, nephrotoxicity, vasodilatation lethargy, and hypersensitivity reactions mediated by histamine release, requiring premedication with corticosteroids and antihistamines [9]. To reduce these side effects’ intensity and so increase Paclitaxel’s safety, better strategies for improving its aqueous solubility are urgently needed. Moreover, even were micronization and/or co-solubilization to improve Paclitaxel’s solubility, a method for selective delivery of this small molecule to tumor cells is equally important, to prevent its quick distribution to healthy cells in all organs immediately after administration.

Due to the side effects of both anticancer drugs and their co-solutes, a critical need exists for improvements in such drug pharmaceutical properties as solubility, stability, and distribution. Toward this end, many drug delivery systems have been developed, including: (1) lipid based drug delivery systems, such as liposomes, niosomes and solid lipid particles; (2) nanotechnology-based drug delivery systems, including micelles, fullerenes, dendrimers, carbon nanotubes, quantum dots, metal-based nanoparticles, and nanofibers; and (3) polymeric delivery systems, including polymeric micelles, polymeric vesicles, polymer protein or polymer small drug conjugates [10].

**Figure 1:**
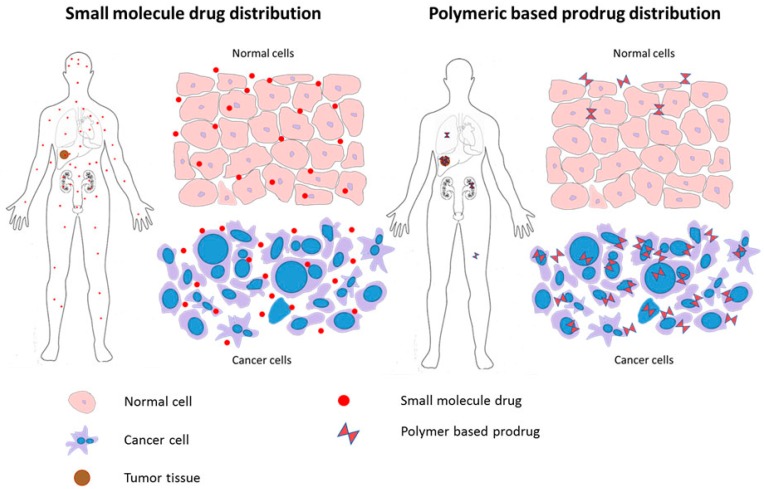
Small molecule drugs are distributed not only in tumor tissue, but are also widely distributed in all healthy tissues, resulting in adverse side effects (**left**); The polymeric macromolecular carrier is designed to spare healthy tissue from toxicity by preferentially accumulating within tumors, primarily by exploiting their disorganized tumor vasculature and poor lymphatic drainage through the enhanced permeation and retention (EPR) effect (**right**).

At administration, antitumor drugs are quickly distributed in the body, exhibiting fast renal clearance, short durations of action, and considerable dissipation, with only a fraction reaching cancer cells. By conjugating these small molecule agents with a high molecular weight polymer, an increase in their solubility and bioavailability can be attained [11] so as to slow renal elimination, lengthen construct duration in blood circulation and improve the construct’s pharmacokinetic profile. A further contribution of such water-soluble macromolecular carriers lies in their ability to facilitate a more targeted drug delivery by exploiting the disorganized vasculature and poor lymphatic drainage that characterizes tumor physiology. This targeting occurs through a phenomenon collectively known as the enhanced permeation and retention effect (EPR) Figure 1. [12]. Exploiting the EPR effect for tumor targeting in this way permits polymer-drug conjugates to be applied at higher doses than possible for any free drug, as polymer-bound drugs will preferentially accumulate and release within tumors. With more drug delivered specifically to a tumor, drug toxicity and side effects will be reduced, and overall therapeutic outcomes improved.

Advances in polymer science have resulted in the synthesis and design of polymers with unique properties. While once confined to roles as co-solutes or mechanical scaffolds, by which to stabilize and solubilize drugs or to control drug release, respectively, polymers now have sophisticated and advanced properties that can be engaged for the development of novel drug delivery vehicles. Prime examples of these advances can be seen in the recently developed synthetic and genetically engineered polymers that can respond to changes in such environmental conditions as pH or temperature. These new constructs currently underlie active research in the development of innovative approaches for active tumor cell targeting [13,14,15].

To optimize their potential as drug carriers, polymers must be well characterized, reproducibly synthesized in quantities, and of a purity acceptable for clinical application. They should also contain the appropriate functional groups needed for coupling or incorporation with specific drugs. Not least, they should be composed of biocompatible, non-immunogenic, and biodegradable materials, remain stable in circulation, but readily release their chemodrug cargo intratumorally or intracellularly. Polymer drug carriers should also maintain a low variability in particle size distribution to ensure the homogeneity of polymer-drug conjugates. By their potential to meet these requirements, synthetic, natural and genetically engineered protein polymers can each contribute urgently needed properties to contemporary drug delivery technology.

Synthetic polymers are human-made polymers, usually fabricated with organic solvents such as polyethylene glycol or *N*-(2-Hydroxypropyl) methacrylamide [16]. Although the structure of synthetic polymers can be easily modified to permit their wide use in drug delivery, problems associated with toxic degradation products or residual toxic components that arise during the process of chemical synthesis have been reported [17]. Natural polymers include polysaccharides, such as dextran and chitosan, polypeptides, and polynucleotides. The use of these polymers as drug-delivery agents is widely reported in cell culture and preclinical trials; however, they have not yet reached clinical trials despite offering greater biocompatibility and biodegradability than synthetic polymers. Genetically engineered polypeptides, developed for controlled and targeted drug delivery, offer polymers with uniform composition and precise molecular weight as compared with chemically synthesized products. These constructs are currently under further development within preclinical trials [15].

In summary, cancer therapies that rely on the systemic administration of anticancer agents face considerable obstacles, with poor solubility main among them. This insolubility, through the consequently lowered bioavailability of chemotherapeutic agents, has to date frustrated treatment efforts and led to the taxing outcome of moderate cancer treatment efficacy. The coupling of antitumor drugs with polymeric drug carriers, whether synthetic, natural or genetically engineered, can help overcome agent insolubility, rendering cancer treatment more effective. These polymers can potentially provide vehicles through which to improve application and target delivery of small, poorly water-soluble chemodrugs so as to improve therapeutic effectiveness and reduce the toll of treatment for cancer patients.

## 2. Classification of Polymers

### 2.1. Synthetic Polymers

The advancement of modern polymer chemistry has allowed the modification of polymer structures, rendering “a tailored polymer for the need” possible. For this reason, a wide variety of synthetic polymers, characterized by variations in the main chain as well as in side chains, are currently available (Table 1).

#### 2.1.1. Polyethylene Glycol (PEG)

**Figure 2:**
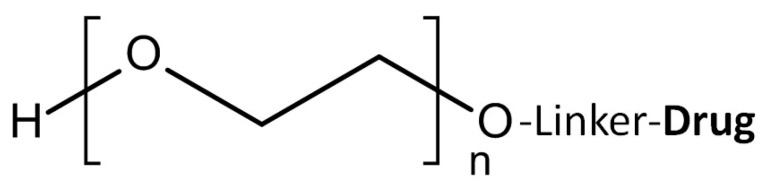
Structure of polyethylene glycol (PEG) and its drug conjugation.

One of the most well-known synthetic polymers, polyethylene glycol (PEG), is a linear polyether compound with ethylene glycol repeats. PEG terminates with hydroxyl group, and can readily be covalently conjugated to many peptides, proteins or drugs (Figure 2). For the coupling of PEG and drugs, agents such as dicyclohexyl carbodiimide (DCC), 1-ethyl-3-(3-dimethylaminopropyl) carbodiimide (EDC), or *N*-hydroxysuccinimide (NHS) esters are commonly used.

The incorporation of PEG with a drug has the advantage of increasing solubility and therefore bioavailability of the resulting conjugates. Additionally, immunogenic responses upon exposure to peptides/proteins may be diminished by the presence of PEG, which can prevent exposure of the peptide’s epitopes and thereby mask peptide/protein recognition by the reticuloendothelial system (RES) [18,19]. The main problem with using PEG, however, lies in its poor drug-loading efficiency, with the limited number of reactive groups in PEG often restricting its broad applications as a drug carrier [20].

**Table 1:** Recent applications of synthetic polymers in prodrug delivery.

Polymer	Application	(co)Polymer	Prodrug	Linker(bond)	Ref.
PEG	Gene delivery	Polyethylenimine(PEI)-PEG	cDNA of herpes simplex virus thymidine kinase gene (HSVtk) and granulocyte–macrophage colony-stimulating factor (GM-CSF)	N.D.	[21]
	Tumor targeting	octreotide(Phe)-PEG	Paclitaxel		[22]
	Improved stability and intracellular drug release	methoxy PEG*-b*-(poly(2-(diisopropylamino) ethyl methacrylate-co-aminopropyl methacrylamide) (PEDPA)	Cis-aconityl-doxorubicin		[23]
	Tumor targeting	PEG	Fusion toxin consisting of the anti-EpCAM DARPin Ec1 and a domain I-deleted variant of ETA (ETA″)	rhinovirus 3C model protease—cleavage linker	[24]
	Drug delivery	3,3′-dithiodipropionic acid functionalized PEG-*b*-poly(l-lysine) (mPEG-*b*-P(LL-DTPA))	Paclitaxel	Disulfide	[25]
	Multidrug resistance	D-α-tocopherol PEG succinate (TPGS)	Paclitaxel	Disulfide	[26]
	Improved the therapeutic efficacy	β-CD, PEG	Doxorubicin	hydrazone	[27]
	Tumor targeting	PEG	Paclitaxel		[28]
	Multidrug resistance	PEG-poly(d,l-lactide)	4-(N)-stearoyl Gemcitabine		[29]
	Theranosis	PEG-polylactic acid (PEG-PLA) l	Dicyanomethylene-4H-pyran-S-CPT	Disulfide	[30]
	Tumor targeting	PEG monomethyl ether (mPEG)	Artesunate	Ester	[31]
	Tumor targeting	PEG monomethyl ether	Camptothecin	Disulfide	[32]
	Tumor targeting	PEG	Camptothecin	Disulfide	[33]
	Tumor targeting	PEG2000	Paclitaxel	MMP2-cleavable linker	[34]
	Nanogel	PLGA-PEG-PLGA	PEGylated Taxol		[35]
	Drug delivery	mPEG-*b*-P(ATMC-co-DTC)	Doxorubicin	Hydrazone	[36]
HPMA	improving anticancer therapy	(mPEG_5000_-*b*-p(HPMAmLac*_2_-r-*AzEMA)	Doxorubicin-glucuronide prodrug (DOX-propGA3)	Glucuronide (β-glucuronidase cleavable linker)	[37]
	Prevent metastasis	HPMA copolymer	E-selectin binding peptide (Esbp)-doxorubicinor (KLAKLAK)_2_		[38]
	Drug delivery	HPMA copolymer	H1-S6A, F8A peptide	GFLG (Cathepsin cleavage linker)	[39]
	Tumor targeting	HPMA copolymer	Doxorubicin	GFLG and MMP cleavable linker	[40]
	Improved the therapeutic efficacy	HPMA copolymer	Doxorubicin, 5-FU	Hydrazone, GFLG	[41]
	Theranosis	Star polymer: poly(amido amine) (PAMAM) dendrimers and HPMA	Doxorubicin or TAMRA fluorescent dye	Hydrazone	[42]
	Improved Bioavailability	Star polymer: poly(amido amine) (PAMAM) dendrimers and HPMA	Pirarubicin	Hydrazone	[43,44]
	Drug delivery	HPMA copolymer	Iodine-125	Hydrazone	[45]
	Drug delivery	HPMA copolymer	Paclitaxel, Gemcitabine	GFLG	[46]
	Drug delivery	Starch + HPMA copolymer	Camptothecin		[47]
	Gene delivery	galactosylated 2-hydroxypropylmethacrylamide-*s*-3-guanidinopropyl methacrylamide (HPMAs–GPMA)	shRNA		[48]
	Drug delivery	HPMA	Indium-111, Yttrium-90		[49]
	Improved the therapeutic efficacy	multiblock poly HPMA	Gemcitabine, Paclitaxel and Doxorubicin	GFLG	[50]
	Theranosis	HPMA copolymer	Zinc protoporphyrin		[16,51]
SMA	Photodynamic therapy	SMA	Zinc protoporphyrin	Amide	[52]
PLGA	Improved Bioavailability	PLGA	Gemcitabine	Amide	[53]
PGG	Improved Bioavailability	PGG	Paclitaxel	Glutamic acid	[54,55]

In spite of these limitations, PEG has been used to deliver a variety of chemical, peptide and gene therapies. For example, Chen *et al.*, produced a Paclitaxel prodrug conjugated to poly (ethylene glycol)-b-poly (l-lysine) by connecting the drug to the polymer via a disulfide bond. This allowed the Paclitaxel to be released from the polymer conjugates when reduced by glutathione, widely utilized as the ideal trigger in redox-responsive delivery systems [25]. Other examples are found in the study of Cai *et al.* [56]. They modified PEG with functional dendrimers to fabricate linear-dendritic copolymers with multiple reactive groups, increasing the number of reactive groups in PEG-based polymers and improving the treatment efficacy of the conjugated drugs [56]. Xia *et al.* also developed a strategy conjugating PEG with multi-numbers of drugs, such as paclitaxel and cisplatin, to maximize the treatment efficiency of the drug-polymer conjugates [57]*.*

#### 2.1.2. The *N*-(2-hydroxypropyl) Methacrylamide (HPMA) Copolymer

**Figure 3:**
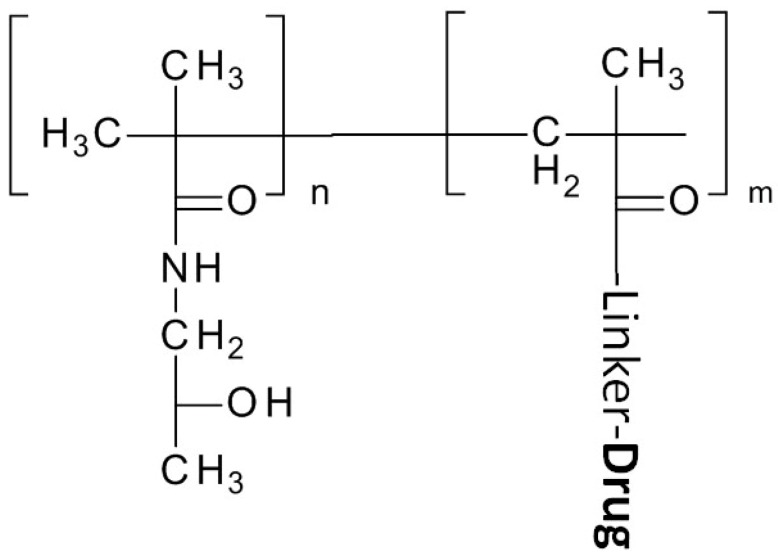
Structure of *N*-(2-hydroxypropyl) Methacrylamide (HPMA) and its drug conjugation.

After early uses of a HPMA monomer/homopolymer as a plasma expander, HPMA’s conjugation with doxorubicin in 1994 clinical trials [58] to its extensive application and investigation for cancer treatment, especially as a copolymer. HPMA, like PEG, is a water soluble, biocompatible, non-immunogenic polymer. Unlike PEG, however, the large number of pendent functional groups in HPMA copolymers permits the conjugation of many drugs (Figure 3). Further, HPMA facilitates activating or releasing the compound from the polymer backbone within a targeted region by manipulation of their bond, a degradable linker. For example, Huang *et al.* used a GFLG cathepsin cleavable linker to deliver a c-Myc inhibitory peptide, the H1-S6A, F8A peptide. An increase in nuclear drug accumulation and an inhibition of tumor growth were achieved with a construct composed of a nuclear localization sequence (NLS) and the polymer complex (R8NLS-HPMAcoPolymer-GFLG-H1-S6A, F8A) [39]

In another study, Etrych *et al.* applied an acid-sensitive hydrazone linker to bridge the HPMA-poly (amide amine) (PAMAM) dendrimers copolymer [42]. In this study, a fluorescent dye, TAMRA, was also conjugated to this dendrimer to visualize the feasibility of the polymer carrier by *in vitro* optical imaging.

#### 2.1.3. Poly (Styrene-Co-Maleic Acid/Anhydride) (SMA)

**Figure 4:**
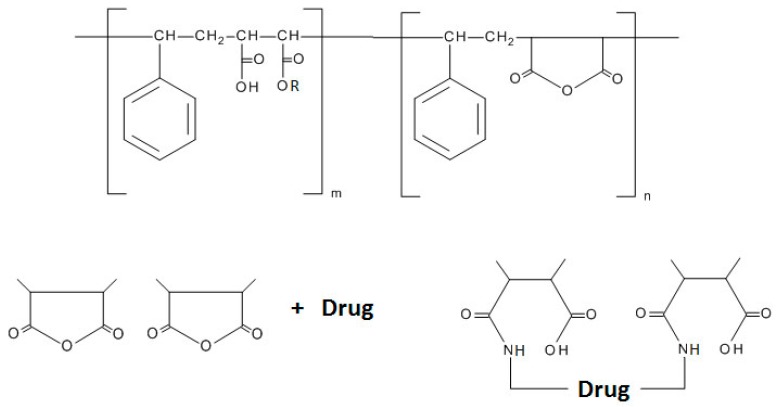
Structure of Poly (styrene-co-maleic acid/anhydride) (SMA) and its drug conjugation [61].

Poly (styrene-co-maleic acid/anhydride) (SMA) is an alternating copolymer composed of styrene and maleic anhydride (Figure 4). Since SMA forms micelles with a hydrophobic styrenic core and a hydrophilic maleic acid surface, a stable and rate-controllable release of micelles can allow surface modification for use in tissue targeting [59]. Although research on SMA is not as active as that for PEG or HPMA, studies have continued to aim at producing prodrugs using this polymer. In 1985, Maeda *et al.* developed a conjugate between SMA and neocarzinostatin (SMANCS) by using an amide bond between the peptide’s terminal amino group and the SMA carboxylanhydride [60]. This work yielded a decreased clearance rate and an increase in the tumor concentration of neocarzinostatin. The enhanced bioavailability and tumor accumulation achieved by this conjugate led to the effective treatment of a variety of solid tumors [61,62]. Recently, Maeda *et al.* developed a styrene-maleic acid-copolymer conjugated with ZnPP (SMA–ZnPP) for photodynamic therapy [63]. In addition to increased tumor accumulation achieved through the EPR effect, light irradiation increased the complex’s cytotoxicity up to six fold.

#### 2.1.4. The Polyglutamic Acid Polymer

**Figure 5:**
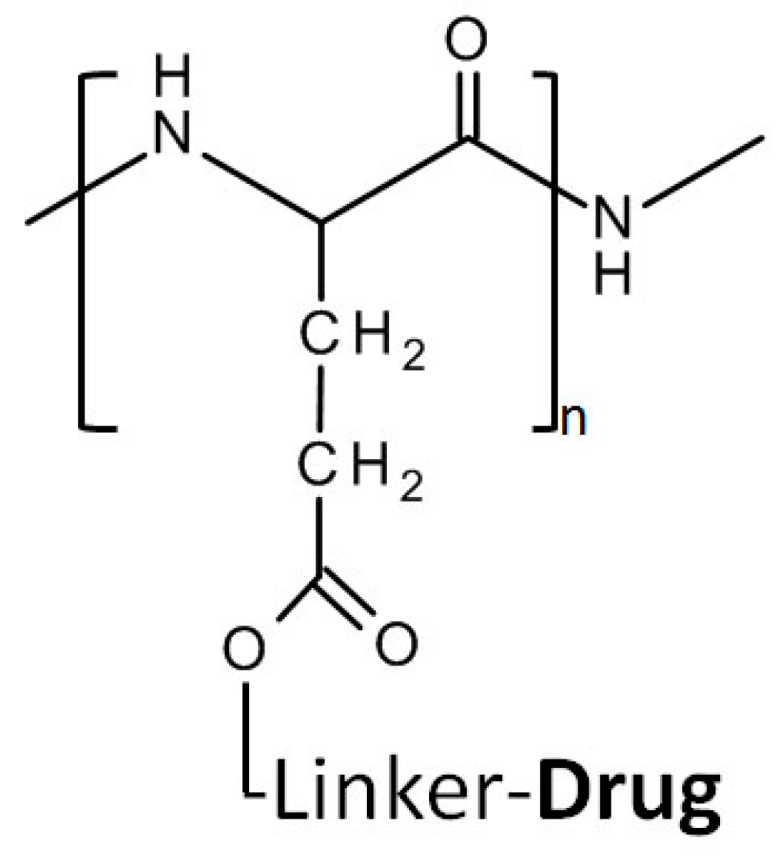
Structure of PGA and its drug conjugation.

Bae *et al.* [64] developed a polymer nanogel based on poly (γ-glutamic acid) (γ-PGA) (Figure 5). Poly (γ-glutamic acid) (γ-PGA), a highly anionic polymer which exhibits excellent biocompatibility and non-cytotoxicity, is naturally synthesized in microbial species, especially *bacilli*. Bae and colleagues developed a thiolated γ-PGA nanogel for doxorubicin delivery that, when used to treat MCF 7 breast cancer cells, showed controlled drug release behavior and higher toxicity as compared to free doxorubicin. Their approach suggests that thiolated γ-PGA nanogel may be a promising drug delivery vehicle in anticancer therapy [64]. Furthermore, in the study of Yu *et al.* [54], the PGA was added with another glutamic acid to each glutamic acid in the polymer backbone producing a poly(l-γ-glutamyl-glutamine)(PGG). This modification shows additional hydrophilicity on a PGG-paclitaxel conjugate as well as improved paclitaxel-loading efficiency in comparison to PGA-paclitaxel conjugates.

Seth *et al.*, [65]used poly (γ-glutamic acid) (γ-PGA) in a mouse melanoma tumor model to deliver both the immuno-stimulating agent, toll-like receptor-7 (TLR-7) agonist-imiquimod and the chemotherapeutic agent paclitaxel. These water insoluble therapeutic agents formed crystalline microstructures that were dispersed in the IgPGA matrix. When the complex was administered by intra-tumoral injection, this combined treatment caused synergistic tumor regression as compared with effects observed when each of the compounds was used alone. This study demonstrates an excellent example and practical approach for the use of water soluble polymers, such as poly (γ-glutamic acid), in formulating micro-dispersions and delivering combinations of water insoluble drugs [65].

#### 2.1.5. Poly (Lactic-Co-Glycolic Acid) (PLGA)

**Figure 6:**
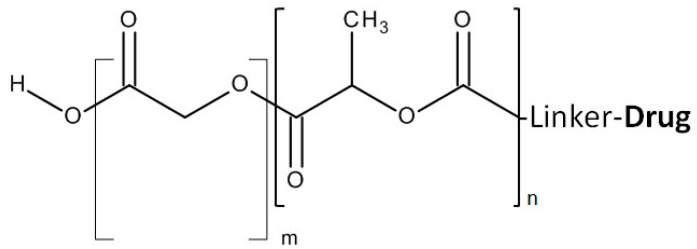
Structure of PLGA and its drug conjugation.

PLGA is a FDA approved biodegradable and biocompatible copolymer of poly lactic acid (PLA) and poly glycolic acid (Figure 6) [66]. This copolymer has been conjugated to gemcitabine via amide bonding [53]. Through the biodegradability of PLGA, the conjugated gemcitabine and released from the complex slowly and over time. Stability and *in vitro* efficacy testing have shown that PLGA-gemcitabine complex has advantages over free Gemcitabine.

To further improve their drug delivery properties, synthetic polymers have often been combined with natural polymers. The synthetic polymer polyethylene glycol (PEG) and the natural polymer chitosan have been applied as conjugates in both *in vitro* and *in vivo* studies*;* however, the secondary stability and short half-life of chitosan-based micelles has hampered outcomes under *in vivo* conditions.

Emami *et al.* [67] used chitosan (see Natural Polymers, below) to develop a novel polymeric micelle with which to deliver paclitaxel. The new micelle, a tocopherol succinate–chitosan–polyethylene glycol–folic acid (TS–CS–PEG–FA), loaded with paclitaxel, yielded both an increased PTX accumulation in tumor tissue and increased toxicity to cancer cells as compared to free PTX. The increased tumor accumulation and favorable pharmacokinetics and tissue distribution observed in this study shows that chitosan-derived micelles have the potential to serve as an effective drug delivery vehicle for paclitaxel [67].

### 2.2. Natural Polymers

Natural polymers are commonly classified in three major classes: polysaccharides, polypeptides and polynucleotides. As polynucleotides are not used as drug carriers in anticancer therapy, only polysaccharides and polypeptides will be discussed below. For a discussion of polynucleotides in cancer therapy, however, interested readers are pointed to the work of Cheng *et al.* [68], Gorska *et al.* [69], and Xiang *et al.* [70].

Polysaccharides and polypeptides can be successfully conjugated with most anti-tumor drugs. Moreover, some of these polymers themselves possess antitumor characteristics. For example, chitosan, through such physical and chemical properties as small particle size and positive surface charge, exerts antitumor activity by disrupting the tumor cell membrane and thereby inducing apoptosis. The results of *in vitro* and *in vivo* studies that used chitosan as an anticancer agent against sarcomas and hepatocarinomas have furthered the view that chitosan can provide a promising antitumor agent, advancing its progress toward clinical trials [71,72].

Natural polymers can now be exploited as a foundation to which poorly soluble drugs can be attached and delivered to tumor sites. Through their considerable versatility the use of these polymers can occasion less drug loss and lower toxicity to healthy tissue.

#### 2.2.1. Chitosan

**Figure 7:**
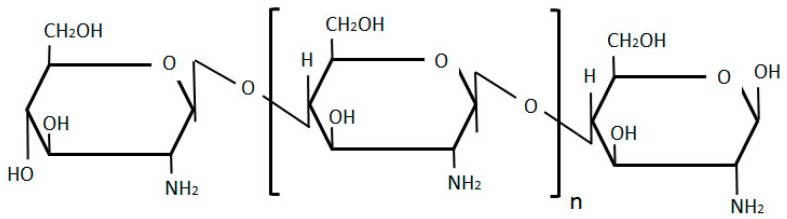
Chitosan structure, composed of deacetylated β-(1-4)-linked d-glucosamine units and of acetylated *N*-acetyl-d-glucosamine units.

Chitosan, a linear polymer composed of interchangeably organized, deacetylated β-(1-4)-linked D glucosamine units and of acetylated *N*-acetyl-d-glucosamine units (Figure 7), is widely used in drug delivery systems. The sole polysaccharide polycation used as a polymer, chitosan binds to negatively charged moieties[20]. Recently, Nogueira and colleagues produced chitosan nanoparticles loaded with the anticancer agent Methotrexate (MTX) [73]. This poorly soluble anticancer agent is extremely potent, but poses serious risks of adverse effects to normal cells, including kidney failure, neurotoxicity and mucositis [73,74,75].

To overcome chitosan’s toxicity and poor solubility, Nogueira *et al.* [73] constructed chitosan-based nanoparticles encapsulating methotrexate (MTX–CS–NPs). Next, to make the chitosan-based nanoparticles pH responsive, these researchers added the amino acid based amphiphile surfactant, 77KS. The incorporation of this pH-sensitive moiety permitted a pH-dependent, controlled release of Methotrexate. *In vitro* experiments were conducted in MCF7 breast cancer cells, with promising results. Future *in vivo* studies will provide more information about the clinical potential of chitosan-based nanoparticles.

Another promising approach using grafted-chitosan polymeric micelles carrying two drugs was developed by Nam *et al.* [76]. The chitosan derivative O-carboxymethyl chitosan (OCMCh), with enhanced solubility, was conjugated first with α-tocopherol, forming α-tocopherol *O*-carboxymethyl (TOC). This chitosan polymer was then ligated with doxorubicin and an anti-human epidermal growth factor receptor 2 [HER2] target peptide, producing the final construct, HPTOC–DOX polymeric micelles. An *in vitro* study that employed this construct against the SK-BR-3 cell line showed a synergistic effect of tocopherol and dox. Furthermore, in an *in vivo* study with SK-BR-3 tumor bearing mice, the anti-HER targeting peptide enhanced not only cellular uptake, but also therapeutic efficacy.

The advantages of chitosan include that it is biodegradable and biocompatible, as well as able to efficiently transport polar drugs across an epithelial surface. In addition, chitosan oligomer derivatives of 3–6 kDa are considered relatively nontoxic. Chitosan’s major disadvantage is its limited solubility at physiological pH, although this can be overcome by combining it with other polymers and/or by chemical modification.

#### 2.2.2. Dextran

**Figure 8:**
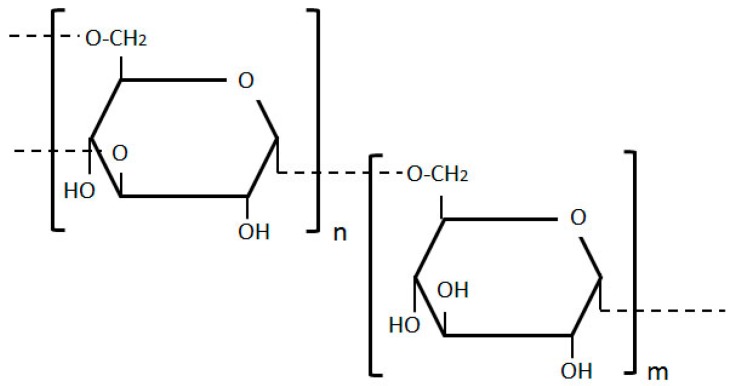
Dextran structure, composed of glucose molecules forming a complex branched glucan chain.

Dextran, structured of glucose molecules that form a complex branched glucan chain of variable length, is a water soluble polymeric saccharide (Figure 8). Dextran-based microspheres have been explored in *in vitro*, *in vivo* and most recently, in clinical trials of breast, colon, hepatic, and pancreatic tumors (please see Table 2. below). These microspheres can satisfy the vast majority of drug carrier requirements. Their primary use in cancer research is to improve the solubility of insoluble anti-tumor drugs.

The slow release systems associated with microspheres have made dextran microspheres (MS) the vehicle of choice for the delivery of mitomycin C (MMC), a promising, potent anticancer agent that functions through bioreductive activation [77]. Many acute as well as chronic toxicities are associated with MMC, however, severely limiting its clinical application. To overcome these impediments and achieve targeted drug delivery to the hypoxic regions of solid tumors, Cheung and colleagues developed a targeted approach composed of oxidized dextran microspheres loaded with MMC [77]. In an *in vitro* model of breast cancer cell-line, EMT6, these researchers applied a doxorubicin-MS therapy, followed by a MMC-MS therapy. This sequential application of two antitumor drugs resulted in a synergistically effective combination of cancer cell killing.

**Table 2:** Current clinical trials of investigational anticancer drugs based on polymer.

Polymer	Name	Drug	Status	Ref.
Dextran	OsteoDex	Alendronate	Phase I	[21,78,79]
	Somadex	Somatostatin	Phase I	[78]
PEG	NK105	Paclitaxel	Phase III	[21]
	NK102	SN-38	Phase II	[80,81]
	NC-6004	Cisplatin	Phase III	[21]
	NC-4016	Dachplatin	Phase I	[21]
	NC-6300	Doxorubicin	Phase I	[21]
poly-l-glutamate	paclitaxel poliglumex, CT-2103	Paclitaxel	Phase III	[82,83]
Cyclodextrin-PEG copolymer	CRLX101	Camptothecin	Phase II	[84]

Dextran microspheres exhibit biodegradability and biocompatibility, as well as being non-immunogenic and nontoxic, all important factors for future clinical applications. These microspheres, easily filtered and neutral, are above all water soluble. However, their clinical translation has been severely hampered by the vexing problem that, when conjugated to antitumor drugs, dextran is rendered immunogenic and non-biodegradable [20]. Other negative effects of dextran polymer use currently include platelet dysfunction, anaphylaxis, and pulmonary, as well as cerebral edema. Studies to determine whether or not these problems can be successfully resolved are now needed.

For nanoparticle assembly, it is important that an anticancer agent/drug and polymer have opposite ionic properties. Therefore, the incorporation of ionic macromolecules with oppositely charged ionic-molecules to form stable nanoparticles can be beneficial [85]. In order to combine advantages and circumvent disadvantages, Lee *et al.* [85] conjugated chitosan and dextran into self-organized nanoparticles for doxorubicin tumor targeted delivery.

#### 2.2.3. Pullulan

**Figure 9:**
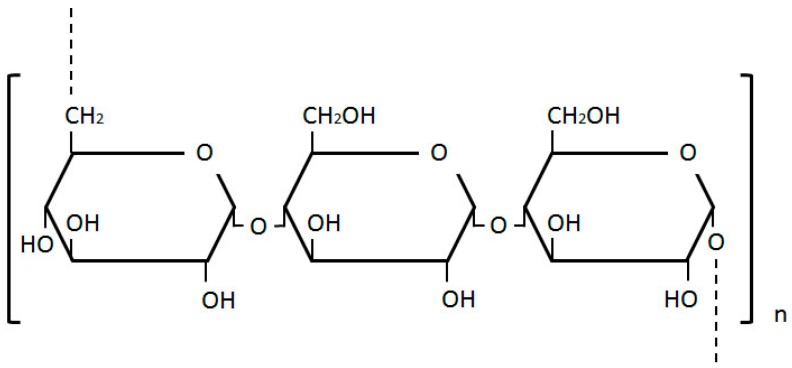
Pullulan structure, composed of maltotriose units.

Pullulan, a natural polysaccharide composed of maltotriose units structured as α-1-4; α-1-6-glucan (Figure 9.) and a liver specific biopolymer [86], is biodegradable, nontoxic, non-mutagenic, and noncarcinogenic. Its low immunogenicity and satisfactory solubility in aqueous and several organic solvents have together led to a range of applications in cancer drug delivery.

Scomparin *et al.* [87] conducted an *in vitro* study with two pullulan bioconjugates. The aim of their study was to design an anticancer polymer therapeutic for targeted tumor cell therapy. The group synthesized the two pullulan derivatives by conjugating pullalan with (1) doxorubicin and (2) doxorubicin and folic acid, with the latter used as a moiety for targeting folic receptors overexpressed in some tumor cell lines. To achieve proper functionality, the pullulan was activated by such further chemical modifications as periodate oxidation and reductive conjugation with cysteamide. Both derivatives, the folic acid-free derivative [(NH_2_ PEG)-Pull-(cyst-Dox)] and the folic acid-doxorubicin-coupled derivative [(FA-PEG)-Pull-(Cyst-Dox)], contained a similar doxorubicin concentration (*w*/*w*), *i.e.*, ~6%, with 4.3% (*w*/*w*) folic acid in (FA-PEG)-Pull-(Cyst-Dox). This group’s *in vitro* study results showed reduced toxicity in the folic acid non-expressing receptor cell line, MCF7, but increased toxicity in the KB cell line, which has over-expressed folic acid receptors. This finding demonstrated the specificity of pullulan polymer bioconjugates and the improved pharmacokinetics of doxorubicin. Moreover, Scomparin and colleague’s design for actively targeting tumors in folic acid over-expressing receptor tumor cell lines, offering a new approach for *in vivo* studies of this bioconjugate pullulan.

The synthesis of pullulan-stabilized gold nanoparticles (PAuNPs) was reported by Ganeshkumar *et al.* [86] for its use in delivering the anticancer drug 5-fluorouracil (5Fu) and folic acid. As described previously, folic acid can be used for active targeting of folate receptor over-expressing cell lines. 5-Fluorouracil was introduced in clinics in 1957 as a poorly-soluble, but potent drug for treating solid tumors. Despite its promise as a chemotherapeutic agent, it showed limited response rates of only 10%–30% [88]. Ganeshkumar and colleagues [86] designed their 2014 study in an effort to increase solubility, stability and specificity, as well as to minimize the side effects of this potent agent. *In vitro* cytotoxicity tests were conducted in free 5-Fu and 5-Fu@AuNPs, as well as in 5-Fu@AuNPs against HepG2 cells with over-expressed folate receptors. Biodistrubution studies in male Wistar rats showed no excessive toxicity in untargeted healthy cells. This group’s encouraging results with 5-Fu@PAuNPs-Fa bioconjugates have set the groundwork for a novel approach in active liver cancer targeting.

Using a similar approach, Zhang *et al.* [89] constructed maleilated pullulan-doxorubicin conjugated with a folate polymeric prodrug for active tumor-targeted delivery, reported as FA-MP-DOX. The purpose of conjugating the construct with folates is to enhance cellular uptake by folate receptor mediated endocytosis. The folate-decorated maleilated pullulan-doxorubicin construct, used to treat ovarian carcinoma A2780 cells, was compared to free doxorubicin and to a maleilated pullulan doxorubicin construct. FA-MP-DOX showed higher toxicity in tumor cells, along with enhanced cellular uptake in ovarian carcinoma A278 cells as compared to free doxorubicin and the MP-DOX construct. These findings suggest that the FA-MP-DOX approach may have potential for clinical application in ovarian carcinoma treatment.

##### Genetically Engineered Polypeptides

Genetically engineered polypeptides have been gaining recognition in biomaterials and drug delivery fields for decades owing to custom design and simple, inexpensive manufacturing. These polypeptides are genetically encoded, providing control over their sequence and molecular weight (MW) to an extent impossible with synthetic polymer analogs. Moreover, these biopolymers are simple and inexpensive to manufacture as they are expressed in *E. coli* at high yields and can be easily purified; their custom design further provides opportunities to confer low immunotoxicity and immunogenicity. These polymers are thus now widely used in anticancer drug delivery to improve the solubility of anti-cancer therapeutics.

#### 2.2.4. Elastin-Like Polypeptide (ELP)

An ELP is a thermally responsive pentapeptide (VPGXaaG) _n_ amino acid repeat, where Xaa is any guest residue amino acid other than proline; the ELP polymer is easily tailored for a wide variety of applications. Depending on an ELP’s primary structure (e.g., amino acid composition), it can form aggregates, hydrogels or micelles in response to stimuli (thermal or pH). As an ELP is a macromolecule, it can exploit the EPR effect to achieve passive targeting to solid tumors. Moreover, an ELP is designed to undergo an inverse transition in response to thermal stimuli. Below the transition temperature, the ELP is disordered and soluble in aqueous solution. At an increase in the solution’s temperature above the transition temperature through application of a mild, external heat, the ELP forms aggregates and can be actively targeted to tumor tissue.

The ELP has now been extensively applied in targeted anticancer drug delivery [90], an area of research that has now expanded to include breast, prostate, ovarian, and brain tumor studies [91,92,93,94]. It has also been used in drug delivery for therapeutic angiogenesis in pre-eclampsia [95] and in biomaterials research [96].

**Figure 10:**
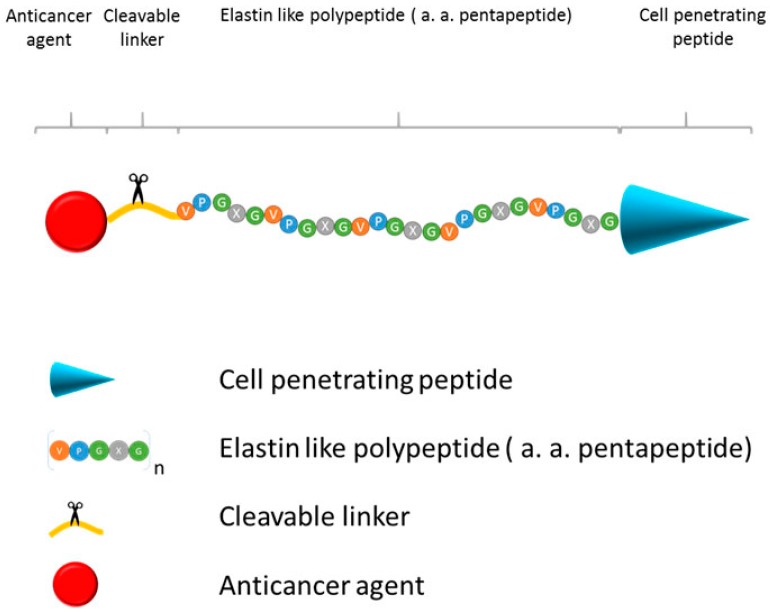
Schematic presentation of Elastin like polypeptide (ELP) polymeric prodrug. Construct consists of (1) Cell penetrating peptide, mediating uptake of ELP in tissues and cancer cells; (2) ELP, thermally responsive biopolymer which can be actively targeted to tumor tissue by external application of mild heat; (3) cleavable linker, releases anticancer agent in tumor tissues and cancer cells, and (4) anticancer agent.

For example, Bidwell *et al.* [97] conjugated the anticancer drug, doxorubicin, to an ELP composed of Tat, a cell penetrating peptide (CPP), ELP and GFLG, a tetrapeptide linker that is a substrate for the lysosomal cathepsin proteases and doxorubicin (Figure 10.). This therapeutic polymeric prodrug was used to treat sensitive MES/SA cells and resistant MES-SA/Dx5 cells. The study’s findings showed that doxorubicin was equally cytotoxic in both cell lines and that ELP-bound doxorubicin accumulated in MES-SA/Dx5 cells, overcoming their drug resistance.

A similar approach was used by Moktan *et al.* [90] to deliver paclitaxel. This polymeric prodrug was composed of three domains: CPP, ELP and paclitaxel. The paclitaxel prodrug contained hydrazine, an acid sensitive linker that is cleaved at lysosomal pH. The study showed that ELP-delivered paclitaxel combined with mild hyperthermia effectively inhibits proliferation in MCF7 and in multidrug resistant MCF7 cells, providing *in vitro* proof of concept for the use of ELP-paclitaxel.

Furthermore, *in vivo* studies support that using ELP as a macromolecular carrier for the delivery of doxorubicin and paclitaxel is a promising approach for clinical application. Moktan *et al.* [92] conjugated an ELP with a CPP (SynB1) at its N-terminus and a 6-maleimidocaproyl hydrazine derivate of doxorubicin at its C-terminus to compare the efficacy of the construct with that of free doxorubicin. Results showed complete tumor inhibition with SynB1-ELP-Dox, as opposed to only moderate tumor inhibition with free dox. Furthermore, the delivery of dox using the ELP construct minimized the cardiac toxicity commonly present with the free form of doxorubicin.

MacKay developed a different approach to delivering Doxorubicin using ELP forming micelles. The ELP polypeptides were modified with short, cysteine-rich segments that were constructed so as to spontaneously self-assemble into 100 nm nanoparticles upon conjugation with doxorubicin. The conjugated doxorubicin nanoparticles exhibited a lower systemic toxicity, but induced almost complete tumor regression in a mouse cancer model [98].

#### 2.2.5. Silk-Elastin Like Polypeptides

A silk-like polypeptide is a genetically engineered polypeptide that is composed of (Gly-Ala-Gly-Ala-Gly-Ser) tandem repeats. For years, silk-like polymers have posed a challenge in that a very low level of their expression is reported when they are derived from the commonly used *E. coli* host [99]. Utilizing substitute hosts that have higher expression levels such as yeast, insect cells, or transgenic plants may result in higher protein yield. However, these alternative routes may negatively affect protein purity. As a result, silk-like polypeptides are not as broadly used as elastin-like polypeptides, whose solubility facilitates numerous paths to more efficient drug delivery.

To make the silk-like polypeptide more suitable for drug delivery, it is often combined with an ELP. Xiao-Xia *et al.* [100] constructed a series of silk-elastin like polymers (SELP) which have the capacity to form micellar nanoparticles upon incubation with doxorubicin using heat as a stimuli. These nanoparticles effectively inhibited proliferation of HeLa cell lines, demonstrating the potential use of drug-loaded SELP for cancer treatment.

## 3. Polymer Drug Conjugates in Clinical Trials

Despite the variety of novel drug targets and sophisticated chemistries available, to the best of our knowledge there have been no FDA-approved small molecule prodrugs based on polymer for cancer treatment yet, while only a limited number of polymer-drug conjugates has been placed in the stage of clinical trials. Currently less than 10 polymer–drug conjugates are on-going in clinical trials (Table 2).

### 3.1. Dextran Conjugates

Through GuaDex technology, a DexTech patented technology platform, dextran is modified and used as the backbone for construction of new candidate medications. OsteoDex and Somadex, which are made from alendronate and somatostatin by DexTech, are being tested in clinical trials to treat castration resistant prostate cancer (CRPC) [78].

### 3.2. HPMA Conjugates

FCE28068, a conjugate of doxorubicin with HPMA copolymer via GFLG linker, showed 5-fold decreased toxicity compared to doxorubicin in a phase I study [101]. In a phase II trial with breast, non-small cell lung, and colorectal cancer patients, FCE28068 had a total of 6 breast and NSCL cancer patients showing partial responses out of 62 patients without severe toxicities [101]. No subsequent reports have been released.

### 3.3. PEG Conjugates

#### 3.3.1. EZN-2208/BEL-0222

SN38 (10-hydroxy-7-ethyl-camptothecin) is a potent topoisomerase I inhibitor and the active moiety of irinotecan (CPT-11). PEG-SN38 is thus a PEGylated conjugate of SN38 for better solubility and an improved pharmacokinetic profile [102]. In addition, the ester bond between the polymer and the drug can be cleaved in PBS, releasing approximately 57% of the conjugated SN-38 from the micelles [80]. PEG-SN38 has been shown to down-modulate HIF-1α involved in tumor invasion, migration, angiogenesis and production of vascular endothelial growth factor (VEGF). PEG-SN38 has produced positive responses in clinical trials for metastatic breast cancer and pediatric neuroblastoma and currently is in a phase III trial [103] 

#### 3.3.2. NC-6300

NC-6300 consists of a PEG polyaspartate block copolymer, an acid-labile hydrazone linker and epirubicin. Preclinical studies using Hep3B/Luc orthotopic hepatic tumor models showed a significant improvement in the survival rate in the group administered NC-6300 at 10 mg/kg compared with that in the group with EPI at 10 mg/kg [81]. Currently, Nano Carrier is conducting a phase I clinical trial in Japan [21].

## 4. Conclusions and Future Direction

Poor solubility and selectivity of anti-cancer drugs limits efficacy of the current cancer treatments. Conjugating drugs to polymers improves drug solubility, pharmacokinetic and pharmacodynamics, as well as increasing their tumor specificity. Thus, polymers provide a remarkable tool through which to increase the therapeutic efficacy of drugs. Despite decades of significant progress in polymer-based drug delivery, the number of FDA approved polymer-based drugs remains very small.

Major objectives in designing new polymer based prodrugs include: (1) improving solubility of the drugs in aqueous solution or lipids; (2) conjugating the drug to the polymer without decreasing its potency; (3) binding the drug tightly to a polymer in circulation, but releasing it inside the tumor cells; and (4) ensuring that the polymer is non-immunogenic, biodegradable and can be cost-effectively produced in sufficient quantities. Successfully addressing these challenges will yield novel and efficient polymer-based prodrugs and establish their significant role in current cancer therapy.
